# Construction of Bovine CypA Gene Expression Vector and Validation of Its Expression in CHO-K1 Cells

**DOI:** 10.3390/ani16030367

**Published:** 2026-01-23

**Authors:** Haidong Liu, Biyu Zhang, Meng Zhou, Yanqiang Zhang, Qian Shi, Haitao Diao, Youfang Gu, Qianqian Hu, Jing Li, Chongmei Ruan

**Affiliations:** 1 College of Animal Science, Anhui Science and Technology University, Chuzhou 233100, China; lhaidong910@gmail.com (H.L.); 15255778563@163.com (B.Z.); 13270885228@163.com (M.Z.); a7a7a7-zyq@163.com (Y.Z.); 13155218780@163.com (Q.S.); diaohaitao0730@163.com (H.D.); guyf@ahstu.edu.cn (Y.G.); 4713412@163.com (Q.H.); 2 Anhui Province Key Laboratory of Animal Nutrition Regulation and Health, Chuzhou 233100, China; 3 Anhui Engineering Technology Research Center of Pork Quality Control and Enhance, Chuzhou 233100, China

**Keywords:** CypA, CHO-K1 cells, eukaryotic expression vectors, bovine mastitis

## Abstract

Bovine mastitis remains a globally prevalent disease, with the limitations of antibiotic-based treatments—such as the rise in antimicrobial resistance and the presence of drug residues—highlighting the urgent need for alternative therapeutic approaches. Inflammation is intricately linked to various cytokines and immunomodulatory proteins, among which cyclophilin A (CypA) serves as a pivotal inflammatory mediator, significantly contributing to the initiation and amplification of inflammatory responses under such conditions. The acquisition of high-purity recombinant protein is a fundamental prerequisite for in vitro functional studies of bovine CypA. This study aimed to construct a eukaryotic expression vector for bovine CypA and verify its expression in CHO-K1 cells. Utilizing the bovine CypA gene sequence available in GenBank, the coding region was artificially synthesized and optimized for codon usage, subsequently being inserted into the pPB[Exp] backbone vector via BsrGI and BstEII double digestion. The resulting polycistronic expression vector contained a CAG promoter driving the CypA transcription, an EF1α promoter driving the EGFP reporter gene, a PGK promoter controlling the puromycin resistance gene, and a C-terminal His-tag. Restriction enzyme digestion and bidirectional Sanger sequencing confirmed that the inserted fragment sequence was completely consistent with the optimized design. Robust EGFP fluorescence was observed 24 h post-transfection and remained stable after puromycin selection. qPCR analysis showed that the Ct value of CypA in the experimental group was 16.20 ± 0.04, while no amplification signal was detected in the control group. Additionally, Western blot analysis identified a CypA-specific band at approximately 18 kDa, confirming the correct expression of the exogenous CypA protein in CHO-K1 cells. Collectively, these results demonstrate the successful construction and validation of a bovine CypA eukaryotic expression vector. The established CHO-K1 expression system exhibited stable and efficient expression, thereby providing a robust foundation for future research on the production and application of recombinant CypA protein.

## Simple Summary

Bovine mastitis is a major disease affecting dairy cow health and milk production, and current treatment options still have important limitations. Previous studies have shown that a protein called cyclophilin A is closely associated with inflammatory responses during mastitis, but further research requires a stable and reliable source of this protein. In this study, we established a method for the stable expression of bovine cyclophilin A in mammalian cells and demonstrated that these cells can continuously express the target protein. This work provides a solid technical foundation for future studies on the role of this protein in bovine mastitis and for the development of related diagnostic or intervention approaches, and it may contribute to improving the prevention and control of bovine mastitis.

## 1. Introduction

Cyclophilin A (CypA) is a ubiquitously expressed intracellular protein and was first identified in bovine thymocytes in the 1980s [1]. It derives its name from its specific binding affinity to the immunosuppressant cyclosporine A. CypA belongs to the cyclophilin family and possesses peptidyl-proline cis-trans isomerase (PPIase) activity, which promotes protein folding, assembly, and transmembrane transport [2]. Subsequent studies have found that CypA is not only present in various mammals such as humans, mice, and cattle, but has also been widely identified in birds, fish, and plants, demonstrating its high conservation and important physiological functions [1,3,4,5]. Beyond its intracellular chaperone functions, CypA can be secreted via non-classical pathways and act extracellularly as an immunomodulatory mediator [6]. As such, it is involved in diverse biological processes, including inflammation, infection, stress, and apoptosis [7,8]. CypA primarily activates signaling pathways such as MAPK and NF-κB by binding to the cell surface receptor CD147, thereby promoting the expression of pro-inflammatory cytokines such as TNF-α, IL-1β, IL-6, IL-8, and MCP-1, and amplifying the inflammatory response [9]. This demonstrates that CypA plays a crucial role in the initiation and amplification of inflammatory responses, and is an important regulatory factor and potential therapeutic target for various inflammatory diseases.

Mastitis represents a significant health challenge for dairy cows and poses a threat to the safety of dairy products. This condition not only markedly diminishes milk yield and compromises the quality of dairy products but also inflicts substantial economic losses on the dairy industry. Currently, clinical treatment for mastitis in dairy cows mainly relies on antibiotics and conventional drugs, but these often come with certain side effects and cannot fundamentally block the continued amplification of inflammatory signals [10]. From a host-centered pathological perspective, in addition to infectious stimuli, oxidative stress has emerged as a critical contributor to mammary gland inflammation in dairy cattle, particularly during metabolic stress, heat stress, and the periparturient period. Recent evidence from ruminant mammary epithelial cell models demonstrated that Cyclophilin A is a redox-responsive protein that is actively secreted under oxidative stress conditions [11]. Research conducted by Satoru Takanashi et al. has revealed that during episodes of mastitis in dairy cows, the secretion level of Cyclophilin A (CypA) in milk is significantly elevated, and this elevation is closely associated with the onset and progression of mastitis [12]. These findings suggest that CypA may serve as a sensitive link between oxidative stress and inflammatory responses in the bovine mammary gland, further supporting its relevance in mastitis-associated pathophysiology. Furthermore, earlier investigations by Satoru Takanashi et al. further demonstrated that extracellular CypA possesses chemotactic activity in cattle, recruiting inflammatory cells to mammary tissue and thereby amplifying the local inflammatory response [13]. This molecular mechanism is widely believed to involve CypA binding to the cell membrane receptor CD147, inducing activation of downstream inflammatory signaling pathways and promoting the expression of pro-inflammatory factors. Transcriptomic studies conducted by Islam et al. have confirmed that in vitro mammary epithelial cell models stimulated by lipopolysaccharide or Staphylococcus aureus exhibit significant upregulation of cytokines such as IL-1α, IL-1β, CXCL3, and CXCL8 upon exposure to exogenous CypA. This directly demonstrates CypA’s role in amplifying pro-inflammatory responses during the immune response to mastitis [14]. Meanwhile, Uemoto et al., in a population genetic analysis of Holstein cows, found that the expression level of the CypA gene in milk was significantly correlated with the level of immunoglobulins, suggesting that CypA has the potential to serve as a genetic marker for mastitis [15]. These findings collectively support the molecular basis for CypA’s role as a key inflammatory regulatory molecule and potential biomarker in bovine mastitis. Therefore, CypA not only has the potential to serve as a biomarker for bovine mastitis but also holds promise as an important target for future anti-inflammatory therapies, particularly in the development of homologous biologics.

Despite the growing recognition of the biological and clinical relevance of CypA in bovine mastitis, most existing studies rely on indirect observations or prokaryotic expression systems, and the availability of homologous recombinant bovine CypA protein produced in a stable mammalian system suitable for downstream functional studies remains limited. In particular, the establishment of a stable mammalian expression system capable of producing biologically relevant bovine CypA protein represents an essential prerequisite for subsequent mechanistic investigations and translational exploration. Therefore, in this study, a codon-optimized bovine CypA eukaryotic expression vector was constructed, and its transcriptional and translational expression was preliminarily validated in CHO-K1 cells; this aims to lay the technical foundation for future research on bovine CypA protein preparation, functional characterization in biologically relevant models, and its potential biological applications in bovine mastitis research.

## 2. Materials and Methods

### 2.1. Plasmid Vectors and Cells

The CHO-K1 cells (Chinese Hamster Ovary-K1) used in the experiment were long-term preserved in our laboratory and routinely cultured in DMEM basal medium containing 10% fetal bovine serum (FBS, Gibco, Waltham, MA, USA) at 37 °C and 5% CO_2_ saturated humidity. The vector backbone was the pPB[Exp] plasmid. The bovine CypA gene (GenBank accession number: XM_002686712.4) was cloned into this vector after codon optimization to form the eukaryotic expression plasmid TSC1041-v1, which was used for subsequent transfection and expression validation of CHO-K1 cells.

### 2.2. Main Reagents and Instruments

The main reagents included fetal bovine serum (FBS, Gibco, Waltham, MA, USA), DMEM basal medium (Hyclone, Marlborough, MA, USA), 0.25% trypsin digestion solution (Beyotime, Haimen, China), puromycin (Invivogen, Toulouse, France), Neon™ transfection system 100 µL kit (Thermo, Waltham, MA, USA), DNA extraction reagent (Solarbio, Beijing, China), AceQ^®^ Universal SYBR qPCR Master Mix (Vazyme, Nanjing, China), RNAprep pure Tissue Kit (Tiangen, Beijing, China), anti-CypA antibody (Boster, Wuhan, China), and HRP-labeled goat anti-rabbit IgG (Boster, Wuhan, China). All other reagents were purchased from Sigma unless otherwise specified.

The main instruments include a biosafety cabinet (Hfsafe-1200LC, Likang, Tainan, Taiwan), a benchtop centrifuge (Sorvall ST16, Thermo, Waltham, MA, USA), a miniature centrifuge (Mini-7K, Ausen, Shanghai, China), a fluorescence microscope (Ts2R/Ts2, NIKON, Tokyo, Japan), a chemiluminescence imaging system (JIAPENG, JP-K900PLUS, Shanghai, China), a cell imaging workstation (EVOS Floid, Thermo), and a single-channel pipette (Thermo).

### 2.3. Construction of Recombinant Eukaryotic Expression Vectors

**Figure 1:**
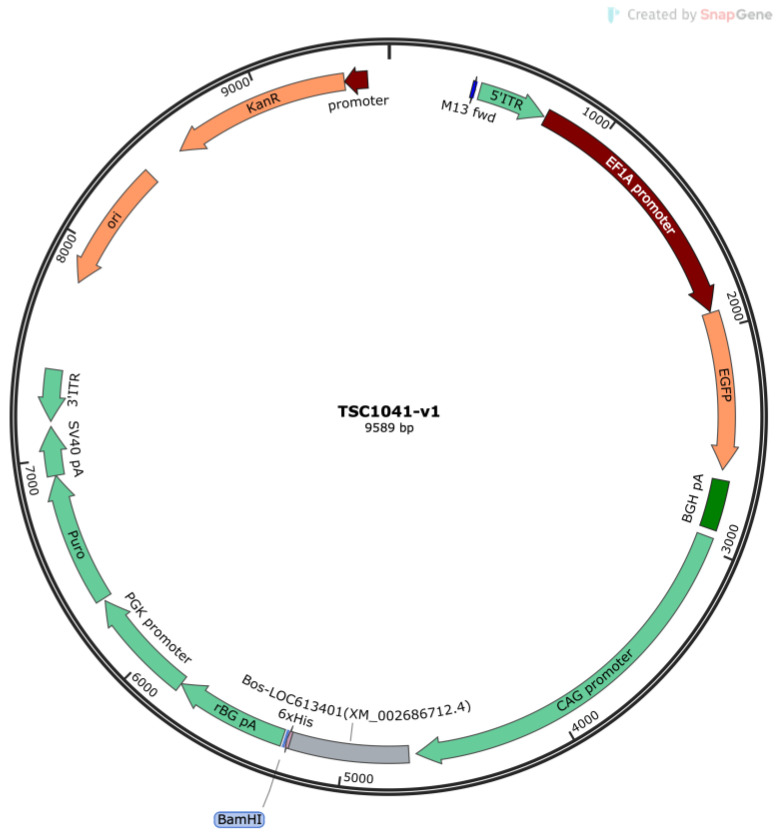
TSC1041-v1 vector map.

To achieve efficient and stable expression of the bovine CypA gene (accession number: XM_002686712.4) in CHO-K1 cells, this study optimized the coding sequence of this gene to suit the codon preferences of CHO cells. The vector used was a polycistronic expression vector, with its core expression elements being an optimized CypA target gene driven by the strong promoter CAG, along with an EF1α-driven EGFP reporter gene and a PGK-driven puromycin resistance gene. For ease of subsequent protein detection and purification, a 6x His-Tag was fused to the C-terminus of the target gene (Figure 1).

The optimized gene fragment was treated with BsrGI and BstEII restriction endonucleases and ligated into the pPB[Exp] backbone vector to construct and obtain the recombinant eukaryotic expression vector TSC1041-v1.

### 2.4. Validation of Recombinant Eukaryotic Expression Vectors

#### 2.4.1. Diagnostic Enzyme Digestion Identification

To preliminarily verify whether the bovine CypA gene fragment was correctly inserted into the pPB[Exp] vector, the recombinant plasmid TSC1041-v1 DNA was double-digested using restriction endonucleases (BsrGI and BstEII). The digestion products were subjected to agarose gel electrophoresis to identify the relative size of the obtained DNA fragments.

#### 2.4.2. DNA Sequencing Alignment

The bidirectional Sanger DNA sequencing was performed by Haixing Biotechnology Co., Ltd. (Suzhou, China). After the sequencing data was assembled, it was compared with the optimized CDS reference sequence.

The optimized CDS sequence was translated into a protein sequence and compared with the original protein sequence corresponding to XM_002686712.4 on NCBI.

### 2.5. Transfection of CHO Cells

CHO-K1 cells were cultured in Dulbecco’s Modified Eagle Medium (DMEM) supplemented with 10% fetal bovine serum and 1% penicillin–streptomycin at 37 °C in a humidified atmosphere containing 5% CO_2_. Cells were passaged at approximately 70–80% confluence.

Healthy CHO-K1 cells were collected, digested with 0.25% trypsin, centrifuged to collect the pellet, resuspended in electroporation buffer, and the carrier was added and mixed thoroughly. Electroporation was performed using the Neon™ transfection system following the manufacturer’s protocol. After electroporation, the cells were immediately transferred to culture dishes containing complete culture medium for re-culture.

### 2.6. Observation of EGFP Fluorescence in Cells

The EF1α promoter-driven EGFP expression cassette in the constructed vector can serve as a reporter gene, allowing the green fluorescence signal of cells to be observed using a fluorescence microscope after transfection and during drug screening.

### 2.7. qPCR Validation of the Target Gene

#### 2.7.1. Primer Design and Synthesis

Primers were designed using Primer Premier 5.0 software based on the corresponding gene sequences in GenBank, with GAPDH as the internal reference gene. The primer sequences are shown in Table 1. The primers were synthesized by Haixing Biotechnology Co., Ltd.

#### 2.7.2. Total RNA Extraction and Reverse Transcription

After transfection and selection, CHO-K1 cells were washed with PBS, and total RNA was extracted using the RNAprep Pure Tissue Kit (Tiangen, Beijing, China) according to the kit instructions. RNA concentration and purity (OD260/280 between 1.9 and 2.1) were measured using a NanoDrop 2000 (Thermo, Waltham, MA, USA). 1 μg of total RNA was used for reverse transcription using a FastKing RT Kit (Tiangen, Beijing, China) to obtain cDNA, which was used as a template for subsequent real-time quantitative PCR.

#### 2.7.3. Real-Time Quantitative PCR (qPCR)

CypA mRNA expression levels were detected using the SYBR Green I fluorescent dye assay. Relative expression levels were calculated using the 2^−ΔCt^ method (ΔCt = Ct_CypA − Ct_GAPDH). Melting curve analysis was performed to verify amplification specificity and the absence of non-specific products. RT-qPCR was used to evaluate the transcriptional expression of the exogenous CypA gene in transfected CHO-K1 cells.

### 2.8. Western Blot Analysis

Stable expressing cells obtained after drug screening were collected and lysed on ice for 20–30 min using RIPA lysis buffer. The lysis buffer was centrifuged at 13,000 r/min for 30 min, and the supernatant was collected and 2× loading buffer was added. Protein samples were denatured at 95 °C for 5 min, separated by electrophoresis on a 15% SDS-PAGE gel, and transferred to a PVDF (Millipore, Burlington, MA, USA) membrane. The membrane was blocked with 5% skim milk at room temperature for 1 h, followed by incubation overnight at 4 °C with anti-CypA primary antibody (1:500, Boster). After washing, the membrane was incubated at room temperature for 1 h with HRP-labeled secondary antibody (1:5000, Boster), and the band signals were detected using ECL chemiluminescence imaging. Western blot analysis was performed to evaluate the translational expression of the exogenous CypA protein in CHO-K1 cells.

## 3. Results

### 3.1. Validation of Recombinant Eukaryotic Expression Vectors

#### 3.1.1. Diagnostic Enzyme Digestion of TSC1041-v1

**Figure 2:**
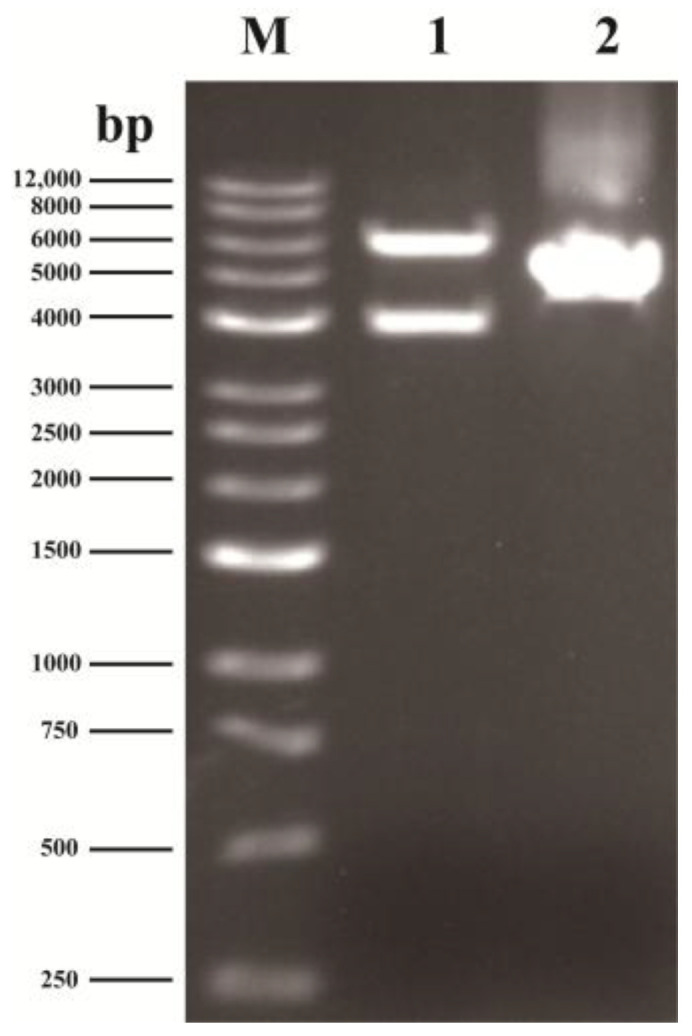
Diagnostic enzyme digestion agarose gel electrophoresis image. (M): DNA marker. (1): TSC1041-v1 digested with BsrGI and BstEII. (2): Undigested TSC1041-v1 plasmid.

The plasmid DNA was digested with BsrGI and BstEII. The agarose gel electrophoresis results showed that the size of the obtained fragments (3830 bp and 5759 bp) was completely consistent with the theoretical expectation, which preliminarily confirmed the correctness of the inserted fragment (Figure 2).

#### 3.1.2. DNA Sequencing Verification

**Figure 3:**
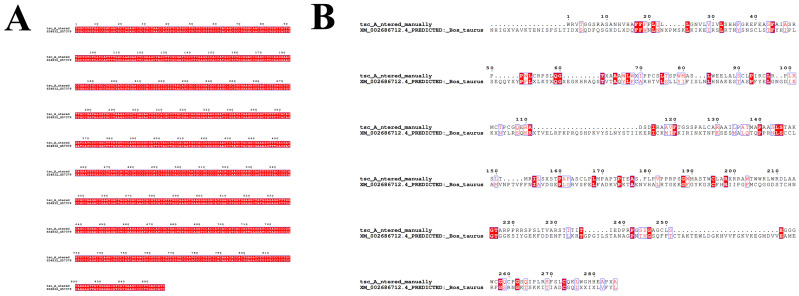
DNA and amino acid sequence alignment diagram. (**A**): Alignment of the sequenced sequence with the CDS DNA sequence. (**B**): Alignment of the coding sequence with the transcript amino acid sequence.

The inserted fragment was subjected to bidirectional Sanger sequencing and DNA sequence alignment for confirmation. The measured sequence was identical to the designed, codon-optimized CDS sequence at the nucleic acid level (Figure 3A). When the translated amino acid sequence was aligned with the reference bovine CypA corresponding to XM_002686712.4, the N-terminal region showed marked homology, whereas several residues in the central and C-terminal regions were divergent (Figure 3B). Thus, the current construct encodes a CypA-related synthetic variant rather than an exact copy of the native bovine sequence.

### 3.2. Observation of EGFP Fluorescence in CHO-K1 Cells

**Figure 4:**
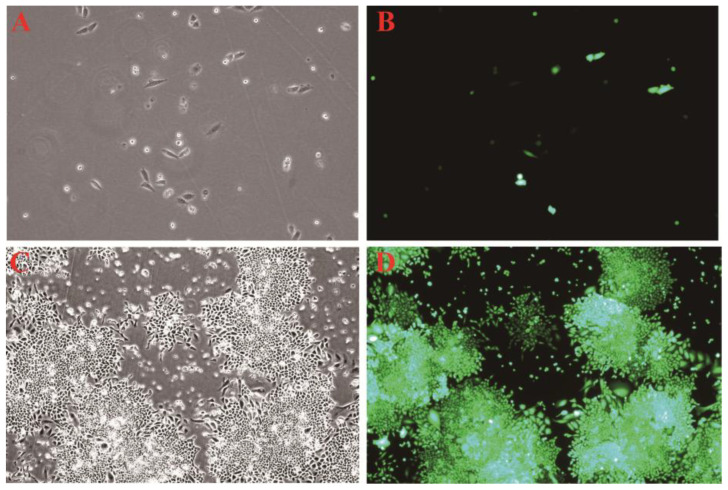
CHO-K1 cell fluorescence observation. (**A**): Brightfield-controlled CHO-K1 cells 24 h after transfection. (**B**): CHO-K1 cells 24 h after transfection. (**C**): Brightfield-controlled CHO-K1 cells 3 days after drug screening. (**D**): CHO-K1 cells 3 days after drug screening.

EGFP fluorescence became detectable 24 h after transfection, corresponding to the cell morphology under bright field (Figure 4A,B). Even after 3 days of selection with puromycin, strong green fluorescence signals were still observed in the cells, with a wide distribution, highly consistent with the cell morphology under bright field (Figure 4C,D). This indicates that the constructed bovine CypA expression vector can be effectively and stably expressed in CHO-K1 cells.

### 3.3. Real-Time Quantitative PCR

To verify the transcriptional expression of exogenous CypA in the stably expressed CHO-K1 cell line, real-time quantitative PCR was performed using cDNA obtained by reverse transcription as a template, and melting curve analysis was used to verify the amplification specificity. The results showed that in CHO-K1-CypA stable expression cells, the Ct value of CypA was 16.20 ± 0.04, while no amplification signal was detected in the control group CHO-K1 cells, indicating that exogenous CypA was efficiently transcribed and expressed in the experimental group.

**Figure 5:**
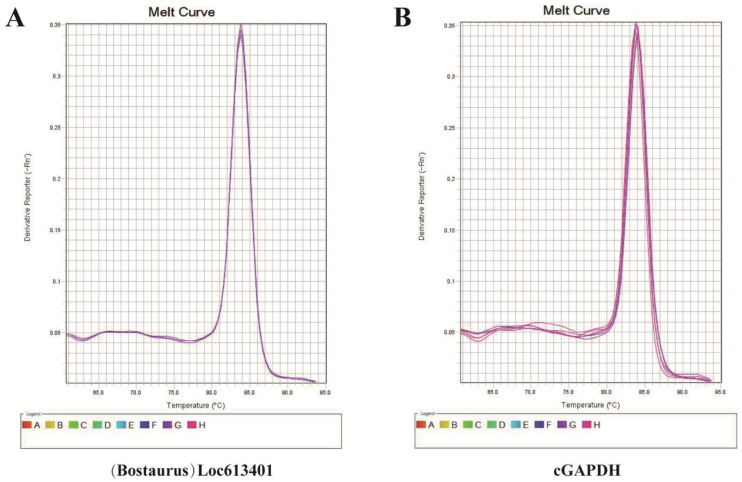
Target gene transcription level. (**A**): CypA melting curve. (**B**): cGAPDH melting curve.

Using GAPDH as an internal control, the ΔCt value in the experimental group was 0.11, indicating efficient transcription of exogenous CypA relative to the internal reference (Table 2). Melting curves showed that both CypA and GAPDH exhibited a single peak, with no non-specific amplification or primer dimers (Figure 5A,B), indicating that the amplification system had good specificity and reliability.

In summary, the qPCR results demonstrate that the expression vector constructed in this study can achieve high-level CypA transcriptional expression in CHO-K1 cells.

### 3.4. Western Blot Results

**Figure 6:**
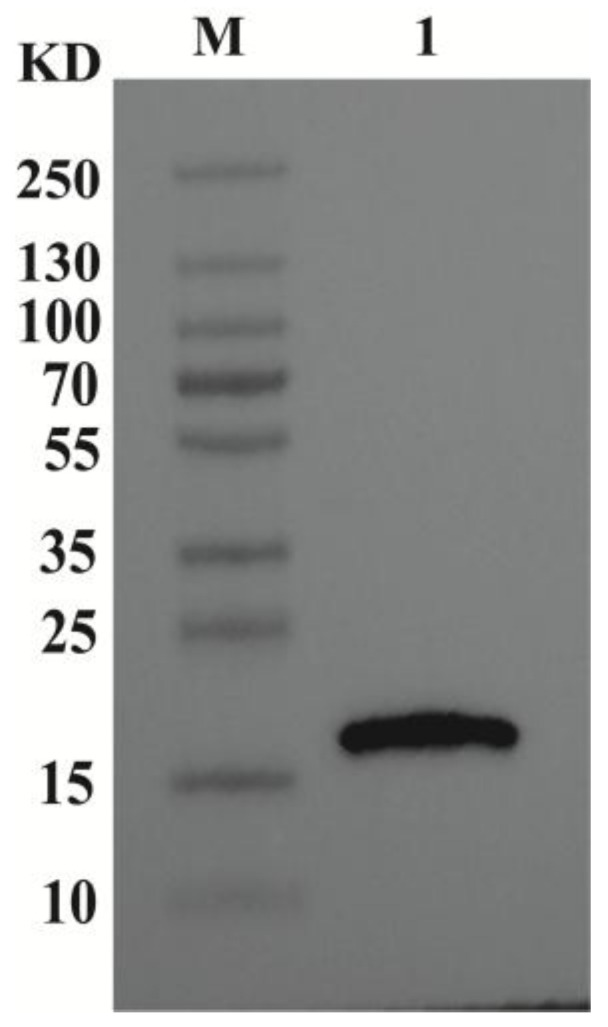
Western blot analysis of CypA protein expression in CHO-K1 cells. (M): Marker. (1): CypA in the recombinant CHO-K1 expression system.

To further verify the expression of CypA at the protein level, Western blot analysis was performed on stably transfected CHO-K1 cells (Figure 6). Western blotting detected a clear CypA band at ~18 kDa, consistent with the predicted molecular weight. This result indicates that the constructed expression vector can achieve correct expression of exogenous CypA protein in CHO-K1 cells, consistent with the aforementioned mRNA level detection results.

## 4. Discussion

Bovine mastitis is a multifaceted inflammatory condition predominantly influenced by the host’s immune response. After pathogens invade the mammary gland, mammary epithelial cells and immune cells recognize pathogen-associated molecular patterns, activating Toll-like receptors and their downstream signaling pathways. This leads to the massive secretion of pro-inflammatory cytokines such as IL-1β, TNF-α, IL-6, and IL-8, thereby mediating neutrophil recruitment and amplification of tissue inflammation. Simultaneously, anti-inflammatory factors such as IL-10 and TGF-β participate in negative feedback regulation to prevent tissue damage caused by excessive inflammation [14]. In both the acute and chronic phases of mastitis, the dynamic balance between pro-inflammatory and anti-inflammatory factors is disrupted by pro-inflammatory cytokines. Excessive release of inflammatory factors not only exacerbates tissue damage but also further activates inflammatory signaling pathways such as NF-κB and MAPK through positive feedback mechanisms [16]. Previous studies have shown that, in addition to traditional cytokines, CypA is also secreted in large quantities during mastitis, suggesting its role as an important inflammatory mediator [12]. As an intracellular molecular chaperone, its main function is to participate in protein folding and transport [17]. In addition, CypA can also be released extracellularly via non-classical pathways, by binding to the cell membrane receptor CD147 [18]. It induces the activation of downstream signaling pathways, thereby further promoting the production of related inflammatory factors [19]. It also enhances the migration and adhesion ability of immune cells [8]. Although previous studies suggest that CypA may participate in the regulation of mastitis-associated inflammatory responses, our understanding of its molecular mechanisms remains limited, particularly at the level of bovine CypA. Notably, CypA is generally regarded as a host-derived inflammatory mediator, and its involvement in mastitis-associated inflammation is more likely linked to common host signaling pathways activated by diverse bacterial pathogens rather than to pathogen-specific mechanisms.

Currently, CypA protein has been extensively studied in the field of human inflammation and immune-related diseases, and recombinant human CypA has been expressed using mammalian expression systems such as HEK293 cells, indicating that eukaryotic platforms are suitable for investigating CypA in human inflammatory and immune contexts. In contrast, research on recombinant CypA protein in animals, especially livestock, remains relatively limited. For example, Wu Qiu et al. cloned the CypA gene from tilapia into the pET-32a vector and successfully constructed and realized its prokaryotic expression system in BL21 [20]; Takanashi et al. also constructed and expressed bovine CypA using the BL21 expression system, obtaining the corresponding recombinant protein [13]. However, when eukaryotic proteins are expressed in prokaryotic hosts, folding abnormalities or inclusion body formation may occur in some cases, affecting their native conformation, enzyme activity, and functional stability. These technical factors may potentially limit the application of recombinant CypA protein in studies requiring high biological relevance. Therefore, establishing a stable and reliable bovine CypA mammalian expression system is of great technical value for obtaining biologically significant recombinant proteins, and also lays a necessary foundation for subsequent functional studies and mechanistic investigations related to bovine mastitis.

This study successfully constructed a bovine CypA eukaryotic expression vector driven by the CAG strong promoter and achieved stable and high-level transcriptional expression of the exogenous gene in CHO-K1 cells. Currently, recombinant eukaryotic protein expression systems often employ HEK293 cells, insect cells, or yeast, but different expression platforms still differ in expression characteristics and applicable scenarios. For example, HEK293 cells are widely used for transient transfection and rapid protein expression screening, whereas their application is more commonly focused on short-term expression rather than long-term stable expression and large-scale production [21,22]. Although insect cell expression systems are characterized by relatively high expression efficiency, their post-translational modification profiles, particularly glycosylation patterns, differ from those of mammalian cells, which may influence the structural properties and biological functions of recombinant proteins [23,24]. Yeast expression systems exhibit substantial differences in protein processing and post-translational modification pathways compared with mammalian cells, which in some cases may limit their suitability for producing recombinant proteins with high biological relevance [25]. In contrast, CHO-K1, a commonly used mammalian cell line, provides a transcriptional and translational environment close to that in vivo, ensuring the correct folding and post-translational modification of exogenous proteins, which is crucial for maintaining the biological function of CypA [26]. Secondly, CHO-K1 has been widely used in the expression and production of recombinant proteins and monoclonal antibodies. It has the characteristics of clear genetic background, high genetic stability and low endogenous interference, which can obtain high-level and stable exogenous gene expression [27,28]. This study obtained stably expressing CHO-K1 cells through puromycin resistance selection, further demonstrating the reliability of this cell line in long-term expression of exogenous proteins. Furthermore, CHO-K1 cells exhibit good expansion capabilities, suitable for both adherent and suspension cultures, facilitating large-scale protein production and subsequent functional experiments [29,30]. Therefore, choosing CHO-K1 as the bovine CypA gene expression host provides a solid experimental foundation for subsequent functional studies.

Building upon this foundation, the stable eukaryotic expression system for bovine CypA established in this study provides crucial technical support for further in-depth research on the mechanisms of mastitis. On one hand, the stable expression system in mammalian cells facilitates the acquisition of recombinant CypA protein with high biological relevance, providing a material basis for future investigations into its potential role in the inflammatory responses of bovine mammary epithelial cells. Using this expression platform, future studies may further explore the mechanisms by which CypA participates in the regulation of inflammatory signaling pathways, immune cell recruitment, and oxidative stress responses by combining it with in vitro mammary epithelial cell models or immune cell models. On the other hand, this expression system also provides a feasible pathway for subsequent purification, structural, and functional analysis of CypA protein, helping to elucidate its potential regulatory patterns in the development and progression of bovine mastitis at the molecular level. Notably, the recombinant CypA obtained based on the mammalian expression system can be used for functional validation under conditions closer to physiological states, providing a reliable platform for future exploration of its potential application as a biological tool and candidate molecular target in mastitis-related research. Therefore, the bovine CypA eukaryotic expression system constructed in this study not only has clear technical significance, but also provides a necessary experimental basis for subsequent basic research and application exploration related to mastitis.

Taken together, the successful establishment and molecular validation of a stable bovine CypA expression system in CHO-K1 cells demonstrate the technical feasibility and robustness of this platform for biologically relevant recombinant protein production in mastitis-related research contexts.

As a technical and methodological study, the present work inevitably has several limitations that should be acknowledged. First, although stable expression of CypA was achieved in CHO-K1 cells, its post-translational modification status, enzymatic activity, and secretory form have not been validated. Second, this study lacks functional assays, such as inflammatory signal transduction, cytokine induction, or protein secretion analysis, to confirm the biological relevance of the expressed CypA in mastitis pathology. Third, the codon optimization process introduced multiple amino acid substitutions in the CypA coding region; therefore, the recombinant protein expressed in this system should be considered a bovine CypA variant, and its PPIase activity and receptor interaction require further characterization in future studies. Future research should focus on investigating the modulation of inflammatory signaling by recombinant CypA in bovine mammary epithelial cells and in vivo mastitis models. Additionally, a systematic analysis of the secretion levels and structural characteristics of CypA should be conducted using protein purification techniques in conjunction with ELISA, Western blotting, and other methodologies. Furthermore, the construction of mutant CypA or CD147-interacting antagonistic peptides based on this vector could further elucidate its molecular mechanisms.

From an application-oriented perspective, the establishment of a stable mammalian expression system for bovine CypA provides a useful technical foundation for future mastitis-related research. Recombinant CypA produced under biologically relevant conditions may serve as a standardized molecular reagent for mechanistic investigations, biomarker-related studies, or immunological assays associated with bovine mammary inflammation.

In addition, the availability of a scalable and well-characterized expression platform offers methodological support for downstream protein production and related exploratory studies, including antibody preparation or proof-of-concept investigations in mastitis research. Although these applications have not been addressed in the present study and remain to be validated, the expression framework established here represents an initial step toward facilitating future translational exploration based on bovine CypA.

## 5. Conclusions

In conclusion, this study successfully established and validated a stable mammalian expression system for bovine CypA in CHO-K1 cells, providing a reliable and biologically relevant platform for recombinant protein production. By integrating codon optimization, rational vector design, and stable cell line construction, this work fills a critical technical gap in bovine CypA research, where previous studies have largely relied on prokaryotic expression systems or lacked standardized eukaryotic platforms. Although the present study is primarily methodological in nature, it establishes a solid foundation for future purification, structural characterization, and functional investigation of CypA in mastitis-related models. Taken together, this work represents a decisive step toward standardizing, scaling, and biologically upgrading the experimental toolkit for bovine CypA research.

## Figures and Tables

**Table 1:** Primer sequence information.

Genes	Primer Sequence	Primer Information	Login ID	Fragment Size/bp
GAPDH	CCAACGTGTCCGTTGTGGAT	cGAPDH-F	NM_001244854.2	171 bp
	AGGTGGAAGAGTGGGAGTCA	cGAPDH-R		
(Bostaurus)LOC613401	TGGTGAATCCCACCGTGTTC	(Bostaurus)LOC613401-F	XM_002686712.4	185 bp
	GCACATAAAGCCGGGGATGA	(Bostaurus)LOC613401-R		

**Table 2:** Relative expression levels of CypA mRNA in stably transfected CHO-K1 cell lines.

Sample Name	①	②	ΔCt (① − ②)	2^−ΔCt^
	Ct (CypA)	Ct (GAPDH)		
CHO-K1-CypA	16.20 ± 0.04	16.08 ± 0.05	0.11	0.93
CHO-K1-Control	Undetermined	18.19 ± 0.17	—	—

## Data Availability

All data generated or analyzed during this study are included in the manuscript. For any additional information or requests, please contact the corresponding author.
